# Editorial and Miscellaneous

**Published:** 1872-04

**Authors:** 


Georgia Medical Association.—The late meeting of the Georgia Medical Association, at Columbus, was altogether the most harmonious and creditable it has been our privilege to attend. The number in attendance was probably the largest which has assembled since its origin, and the papers, in number and character, far exceeded in value those which have been presented at any meeting in the last fourteen years. The discussions were decidedly interesting and profitable, and exhibited marked ability upon the part of a number of those who participated. And finally, as a social reunion, it will long be held as eminent in the good fellowship which abounded among those who had assembled from the various quarters of our widely-extended State, and is specially to be remembered for the munificent hospitality and warm-hearted welcome of the medical profession and citizens of Columbus.

The "Transactions" will contain all the papers which are decided by the Committee on Publication as worthy of a place in the volume; but as some months will probably elapse before its issue, we are pleased to inform our readers that the authors of a number of the most valuable have selected this journal (under a privilege granted by the body) as the medium through which they will be given to the profession.

Dr. Wm. Abram Love.—While traveling for the restoration of his health, Dr. Wm. Abram Love, of this city, has kindly consented to represent the interests of this journal. We sincerely hope that he may return to his home with renewed vigor and energy, and to the prosecution of his physiological and pathological investigations in connection with the liver, kidneys and genito-urinary system, and especially to the successful practical pursuits of his profession.

American Medical Association.—This body meets in Philadelphia on May 7th, and if wise counsels prevail, may still be fruitful of much good to the profession; but, as all admit, it is a crisis in its history, and without the exercise of wisdom and prudence, it will cease to be the *real* representative of American Medicine.

In this latter event, doubtless the best men of the profession throughout the entire country will combine to form a new organization (not necessarily upon the ruins of the other), access to which will not be so readily obtained as in the case of the present Association. We hope Georgia will be largely represented, for which the Association at its recent meeting provided.

Books, Etc., Received.—We have a number of books and pamphlets on hand, reviews of which we had fully designed to insert in this number of the Journal, but have found it impracticable. We hope our friends who have thus favored us will not become impatient, as we shall certainly dispose of everything on hand, of this character, in the May number. We specially regret our inability to notice properly the valuable books received from Henry C. Lea, Philadelphia, which were acknowledged in the previous number, with the exception of the new edition of the "Diseases of Women," by Dr. T. Gaillard Thomas.

Dr. McDowell's Address.—We regret our inability to insert in this number the annual address of the retiring President of the Georgia Medical Association, Dr. G. M. McDowell. We hope, however, to obtain the address, and publish it in the next number.

For urgent reasons, several advertisements and the list of contributors have been left out of this number of the Journal. These irregularities will be arrrnged in the next issue by an advertising sheet, which is now in course of preparation.

Communications and exchanges should be addressed to the Atlanta Medical and Surgical Journal, care of "The Plantation Publishing Company," Atlanta, Georgia.

Dr. E. J. Kirkscey.—We commend to the profession in Arkansas our friend, E. J. Kirkscey, M.D., late Vice-President of the Georgia Medical Association, who has kindly consented to represent the interests of the Journal in that State during a sojourn of some months.

---

